# A Rare Neurological Presentation Post-Cardiac Catheterization

**DOI:** 10.7759/cureus.22948

**Published:** 2022-03-08

**Authors:** Mahsa Mohammadian, Ahmad Damati

**Affiliations:** 1 Internal Medicine, Rutgers-New Jersey Medical School/Trinitas Regional Medical Center, Elizabeth, USA; 2 Department of Cardiology, Saint Joseph University Hospital, Paterson, USA

**Keywords:** oculomotor nerve palsy, isolated midbrain infarction, embolic infarct, aortic valve stenosis, cardiac catheterization

## Abstract

Unilateral Isolated oculomotor nerve palsy is a rare neurological complication after cardiac catheterization. Concomitant thalamus and midbrain infarction secondary to embolic events involving the artery of Percheron after cardiac catheterization have been reported in the literature. However, isolated midbrain infarction is a rare neurological deficit. Here, we present the case of a patient who presented with mild left-sided ptosis, binocular diplopia, and partially impaired left eye adduction two hours after cardiac catheterization. Brain magnetic resonance imaging revealed a focal area of restricted diffusion within the midbrain tegmentum, confirming this rare brainstem stroke.

## Introduction

Cardiac catheterization is a diagnostic and interventional procedure for coronary and valvular heart diseases. Although it is considered relatively noninvasive, as it is usually done under local anesthesia, it can potentially cause various adverse events. One of the well-known complications is embolic stroke. In this article, we report a rare case of binocular diplopia that occurred within two hours of the procedure. Brain magnetic resonance imaging (MRI) was consistent with the diagnosis of isolated midbrain infarction.

## Case presentation

**Figure 1:**
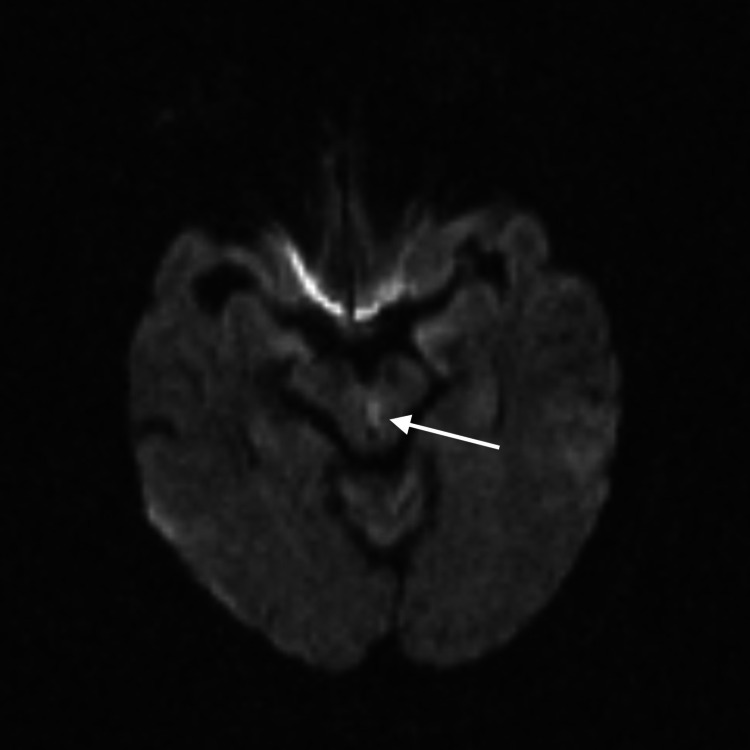
Brain MRI showing a linear area of restricted diffusion within the midbrain tegmentum. MRI: magnetic resonance imaging

A 58-year-old male with a medical history significant for insulin-dependent diabetes mellitus, hypertension, dyslipidemia, cardiac arrest secondary to ventricular fibrillation, coronary artery disease status post-coronary artery bypass graft (CABG) and subsequent percutaneous coronary intervention (PCI), and aortic valve stenosis was admitted in the hospital for cardiac catheterization prior to transcatheter aortic valve replacement (TAVR). The patient had an extensive history of ischemic heart disease that was treated with CABG about nine years before the current presentation. Cardiac catheterization three years after CABG had shown patent left internal mammary artery to the left anterior descending artery, occluded saphenous vein graft (SVG) to diagonal, acute thrombosis of the proximal segment of the SVG to the obtuse marginal (OM) branch of the left circumflex coronary with subsequent successful balloon angioplasty, and drug-eluting stent placement. Later, a recurrent non-ST-elevation myocardial infarction required PCI due to occluded graft. During the last admission, he was diagnosed with severe aortic stenosis and was recommended to proceed with TAVR which was postponed due to recurrent pneumonia. For further evaluation before TAVR, elective cardiac catheterization was scheduled during this admission. On presentation, the patient had stable vital signs and unremarkable physical examination except for a 4/6 ejection systolic murmur in the aortic area. He underwent left heart catheterization which showed patent previous grafts and wall motion abnormality indicative of old localized inferior myocardial infarction, normal pulmonary artery pressure, and severe aortic stenosis with a valve area of 0.57 cm^2^. The patient had received mild sedation during catheterization and was alert, awake, and oriented immediately after the procedure. Two hours later, the patient was noticed to have new-onset diplopia and was evaluated immediately by the physician. He reported having double vision that disappeared while covering either eye. Neurological examination was remarkable for mild ptosis of the left-sided eyelid, binocular diplopia, and partially impaired left eye adduction. Sensory, motor power, and reflexes were intact during the physical examination. No nystagmus was notes. Computed tomography (CT) scan of head was unremarkable for acute infarction or hemorrhage. CT angiography of the head and neck showed 80% calcific stenosis of the left and 20% stenosis of the right internal carotid artery bulb but was otherwise unremarkable. Brain MRI without contrast revealed mild small vessel ischemic changes and a focal area of restricted diffusion within the midbrain tegmentum, which given the patient’s history of diplopia was representing an acute stroke (Figure 1).

The patient was evaluated by a neurologist with the diagnosis of acute stroke. He was not a suitable candidate for tissue plasminogen activator (TPA) administration due to the recent arterial puncture at a non-compressible site. His previous home medications including antiplatelet treatment with aspirin 81 mg and clopidogrel 75 mg, high-intensity statin, and ezetimibe 10 mg oral daily were continued to prevent secondary stroke. He was observed a few more days in the hospital, and at the time of the discharge, his diplopia remained unchanged.

## Discussion

Strokes after cardiac catheterization are uncommon and, most of the time, are silent [1]. The majority of post-cardiac catheterization strokes are ischemic compared to hemorrhagic. Thrombus, calcific, or cholesterol particles can potentially get dislodged during catheterization and can cause embolic infarcts [2]. Larger catheters that are usually used in interventional procedures and a longer duration of the procedure can lead to a higher incidence of neurological events. Predisposing factors for stroke include age more than 75, hypertension, diabetes mellitus, history of a previous stroke, renal failure, intra-aortic balloon pump, congestive heart failure, triple vessel coronary artery disease, a higher dose of contrast, and emergent procedure [3]. As presented, our patient had multiple risk factors. In the literature, the incidence of post-cardiac catheterization stroke has been estimated between 0.03% and 0.6%. Rates of 0.03-0.3% in diagnostic procedures [4] and 0.3-0.4% in post-PCIs have been reported [3]. A recent meta-analysis study on more than 2,000 patients revealed clinical evidence of neurologic deficits in approximately 0.6% of the patients [1].

Retrograde catheterization of the aortic valve in a patient with valvular aortic stenosis has been shown to be associated with an increased risk of cerebral embolic events. Omran et al. conducted a study on 152 patients with valvular aortic stenosis who underwent catheterization with and without aortic valve passage. The study revealed that 22% of the patients with retrograde catheterization had focal diffusion imaging abnormalities, whereas only 3% had clinical stroke evidence [5]. Regarding the location of the neurological deficits, Al-Mubarak et al. reported that most of the post-catheterization embolic strokes are associated with either the common carotid bifurcation or the proximal middle cerebral artery occlusion [6]. Another study revealed middle cerebral artery infarction in 24%, posterior cerebral artery in 19%, basilar artery in 5%, vertebral artery in 10%, and occlusion in two anterior circulation branches in 43% of the patients [7].

Thalamic and midbrain arterial supply can have a complex anatomy. There are a few rare anatomical variants, and one of them is the artery of Percheron. This artery arises from the P1 segment of a posterior circulating artery and supplies both the paramedian thalami and rostral midbrain bilaterally. The incidence of artery of Percheron infarction is very low (0.1-2%) in all ischemic strokes due to variations of paramedian thalamic-mesencephalic arterial supply [8]. Usually, occlusion of the artery of Percheron can result in bilateral thalamic stroke with or without additional midbrain stroke. Patients typically present with altered mental status, vertical gaze palsy, and memory impairment.

In our literature review, a few cases reported artery of Percheron infarction after coronary angiography, where patients presented with thalamic strokes [8,9]. Lazzaro et al. performed a study on 37 patients with artery of Percheron infarction (not necessarily post-cardiac catheterization) during a 10-year period and reported four ischemic patterns of the thalamic and midbrain involvement. An isolated midbrain embolic event was not detected in any of the cases [10]. In this report, we present a unilateral isolated oculomotor nerve palsy, indicative of isolated midbrain infarction. 

To our knowledge, there have been only three cases of post-cardiac catheterization isolated midbrain infarct reported in the literature. In 1984, Biller et al. presented a case of ptosis, bilateral internuclear ophthalmoplegia, retraction nystagmus, and somnolence that developed after percutaneous transluminal angioplasty. Rostral midbrain dorsal tegmental infarction was confirmed with high-resolution CT [11]. The next case report was in 1991 by Liu et al. who reported two patients with bilateral ptosis and oculomotor palsies, with one patient having undergone coronary angioplasty before presentation [12]. Mihaescu et al. presented a patient who developed complete bilateral ptosis, partial up gaze paresis, and right internuclear ophthalmoplegia following cardiac catheterization. Diffusion-weighted imaging demonstrated an increased signal compatible with a diffusion abnormality in the periaqueductal gray matter at the level of the cerebral peduncles eight hours after the procedure. Mild improvement in the patient’s ptosis was noted four weeks later [13].

In this case report, we demonstrated a case who presented with mild left-sided ptosis, binocular diplopia, and partially impaired left eye adduction two hours after cardiac catheterization. Brain MRI revealed a focal area of restricted diffusion within the midbrain tegmentum, representing an acute recent stroke. The patient had minimal improvement at the time of discharge. We believe that isolated oculomotor nerve palsy secondary to isolated midbrain infarction is a rare neurological complication post-cardiac catheterization. Post-stroke restoration of the blood flow is the ideal management that should be done as soon as possible. However, TPA administration is concerning due to the increased risk of bleeding, as one of the exclusion criteria for use of TPA is arterial puncture at a non-compressible site within seven days. Contrarily, there are some data from a retrospective study that showed TPA might be safe and efficacious [14]. The standard management of acute ischemic stroke with antiplatelets and less commonly mechanical thrombectomy of the large vessel occlusion is applied to post-catheterization infarctions as well. Unfortunately, there is not a definite evidence-based guideline regarding the treatment strategy in this condition.

## Conclusions

Although post-cardiac catheterization stroke is rare, it is well known. Retrograde catheterization of the aortic valve in patients with valvular aortic stenosis is associated with an increased risk of cerebral embolic events. Close monitoring for possible neurological deficits, such as gaze palsy, diplopia, and ptosis, and more evident symptoms, such as memory impairment, disturbances of consciousness, and motor/sensory deficits, is crucial. This consideration should be even more prominent in patients with multiple risk factors, and neurological evaluation and treatment should be done promptly.
